# Does taxonomic and numerical resolution affect the assessment of invertebrate community structure in New World freshwater wetlands?

**DOI:** 10.1016/j.ecolind.2021.107437

**Published:** 2021-02-15

**Authors:** Mateus M. Pires, Marta G. Grech, Cristina Stenert, Leonardo Maltchik, Luis B. Epele, Kyle I. McLean, Jamie M. Kneitel, Douglas A. Bell, Hamish S. Greig, Chase R. Gagne, Darold P. Batzer

**Affiliations:** a Universidade do Vale do Rio dos Sinos (UNISINOS), 950 Unisinos av, São Leopoldo, RS, Brazil; b Centro de Investigación Esquel de Montaña y Estepa Patagónica (CONICET-UNPSJB), Roca 780, Esquel, Chubut, Argentina; c Facultad de Ciencias Naturales y Ciencias de la Salud (UNPSJB), Sede Esquel, Esquel, Chubut, Argentina; d U.S. Geological Survey, Northern Prairie Wildlife Research Center, Jamestown, ND 58401, USA; e California State University, 6000 J St, Sacramento, CA 95819, USA; f East Bay Regional Park District, 2950 Peralta Oaks Court, Oakland, CA 94605, USA; g University of Maine, 212 Deering Hall, Orono, ME 04469, USA; h University of Georgia, 120 Cedar St, Athens, GA 30602, USA

**Keywords:** Alpha diversity, Beta diversity, Congruence, Freshwater ponds, Higher-taxon approach, Taxonomic surrogacy

## Abstract

The efficiency of biodiversity assessments and biomonitoring studies is commonly challenged by limitations in taxonomic identification and quantification approaches. In this study, we assessed the effects of different taxonomic and numerical resolutions on a range of community structure metrics in invertebrate compositional data sets from six regions distributed across North and South America. We specifically assessed the degree of similarity in the metrics (richness, equitability, beta diversity, heterogeneity in community composition and congruence) for data sets identified to a coarse resolution (usually family level) and the finest taxonomic resolution practical (usually genus level, sometimes species or morphospecies) and by presence-absence and relative abundance numerical resolutions. Spearman correlations showed highly significant and positive associations between univariate metrics (richness and equitability) calculated for coarse- and finest-resolution datasets. Procrustes analysis detected significant congruence between composition datasets. Higher correlation coefficients were found for datasets with the same numerical resolutions regardless of the taxonomic level (about 90%), while the correlations for comparisons across numerical resolutions were consistently lower. Our findings indicate that family-level resolution can be used as a surrogate of finer taxonomic resolutions to calculate a range of biodiversity metrics commonly used to describe invertebrate community structure patterns in New World freshwater wetlands without significant loss of information. However, conclusions on biodiversity patterns derived from datasets with different numerical resolutions should be critically considered in studies on wetland invertebrates.

## 1. Introduction

The taxonomic impediment (Terlizzi et al., 2009) is a major challenge for biodiversity research (Bevilacqua et al., 2012). Knowledge about the biodiversity of many groups is limited, and the availability of specialized taxonomists to collaborate with ecologists is inconsistent throughout the world (Wheeler et al., 2004; Hortal et al., 2015). Consequently, it has become commonplace for researchers to rely on assessments conducted at lower (coarser) taxonomic resolutions, known as taxonomic surrogacy (or the higher-taxon approach; i.e. lower taxonomic resolution relies on higher taxonomic categories; Bertrand et al., 2006; Bevilacqua et al., 2012), to describe community patterns and assess ecological relationships. Indeed, several studies have shown that community patterns obtained with species level data, including those revealed by multivariate techniques, are still observed at lower taxonomic resolutions (usually the family level) (Kallimanis et al., 2012; Hernandez et al., 2013; Mueller et al., 2013; de Oliveira et al., 2020).

In contrast, several assessments and meta-analyses suggest that the efficacy of the higher-taxon approach can be variable (Lenat and Resh, 2001; Jones, 2008; Mueller et al., 2013; de Oliveira et al., 2020). The similarity in statistic outcomes can be weakened at coarser resolutions for different taxa or vary according to geography or ecosystem type; these inconsistencies include inaccurate estimates of biodiversity patterns (Melo, 2005; Heino and Soininen, 2007; Rosser and Eggleton, 2011; Heino, 2014; Vilmi et al., 2016). Different factors may explain the variation in results when different taxonomic resolutions are used, for example, species-to-higher taxa ratios, spatial extent, methods of data transformation methods and differences in niche conservatism among groups (Bevilacqua et al., 2012; Mueller et al., 2013; Neeson et al., 2013; Rosser, 2017). This lack of generality led to criticism by several authors and to a long-standing debate on the appropriateness of the use of the higher-taxon approach for reliable ecological assessments (Lenat and Resh, 2001; Jones, 2008; Rosser and Eggleton, 2011; Mueller et al., 2013; de Oliveira et al., 2020). While many authors maintain that the species level is the most appropriate resolution to describe ecological responses (Lenat and Resh, 2001; Jones, 2008; Terlizzi et al., 2009; Rosser and Eggleton, 2011), others suggest that the higher-taxon approach is justified by its favorable cost-benefit relationship given the logistic and financial constraints faced by many researchers. The higher-taxon approach is especially appropriate for rapid biological assessments or to prioritize conservation efforts in areas lacking needed taxonomic resources (e.g., keys, experts) (Bailey et al., 2001; Jones, 2008).

The higher-taxon approach has been extensively used for aquatic invertebrates in streams; in these ecosystems, the patterns observed at coarse taxonomic resolutions usually mirror results obtained with finer taxonomic resolutions (Melo, 2005; Heino and Soininen, 2007; Monk et al., 2012; Heino, 2014; Vilmi et al., 2016). It has, however, rarely been investigated for aquatic invertebrates in wetlands (Epele and Miserendino, 2015), despite the diversity of habitat types (Batzer, 2013) and the acknowledged ecological value and threatened status of these ecosystems (Costanza et al., 2014). The taxonomy of fauna dominating streams (e.g. Ephemeroptera, Plecoptera and Trichoptera) is well known, and most studies in streams can thus be based on data with fine taxonomic resolution (genus, species, or morphospecies, Lenat and Resh, 2001). This is rarely an option for studies of wetland invertebrate communities because most invertebrate species in these ecosystems belong to highly diverse insect orders (e.g., Coleoptera, Diptera), for which adults are frequently required to key individuals to species, and sometimes genus. Consequently, community-level assessments of invertebrates in wetlands based on species-level data are usually restricted to particular families or genera (Chessman et al., 2002; King and Richardson, 2002; Chadd and Extence, 2004; Simić et al., 2007; Garrido and Munilla, 2008; McDaniel et al., 2017; Grech et al., 2019).

In addition to the issue of taxonomic resolution, differences in numerical resolution (e.g., relative abundance and presence-absence) can influence the observed patterns of community structure (Melo, 2005; Mueller et al., 2013; Heino, 2014; Sgarbi et al., 2020). Analyses based on presence-absence data sets tend to increase the influence of rarer taxa (Anderson et al., 2011) and may be especially useful for diversity studies. Analyses based on abundance data emphasize the importance of common taxa, and may be especially useful for studying interspecific interactions (Heino 2014). Differences in numerical resolution may constitute an especially important issue for the study of community structure in freshwater wetlands because the invertebrate communities are usually numerically dominated by a few taxa such as chironomids, oligochaetes and microcrustaceans (e.g. Kratzer and Batzer, 2007; Moraes et al., 2014), which are the same taxa that are rarely classified with a fine taxonomic resolution.

In this study, we assessed the effects of different taxonomic and numerical resolutions on a range of metrics commonly used in the characterization of the community structure, focusing on aquatic invertebrates in New World freshwater wetlands. We tested the congruence in community-level patterns between ‘coarse’ (usually family) and ‘fine’ (typically genus) taxonomic resolution and between presence-absence and relative abundance data sets for a range of univariate and multivariate metrics. We analyzed six independent invertebrate data sets from different wetland habitats distributed across subtropical and temperate regions of North and South America. If consistent patterns span across this range of habitats, the application of the higher-taxon approach and the use of presence-absence data could provide a useful shortcut in the assessment of biodiversity and community patterns of wetland invertebrates in the New World.

## 2. Material and methods

### 2.1. Study regions and reference sources

**Fig. 1.**
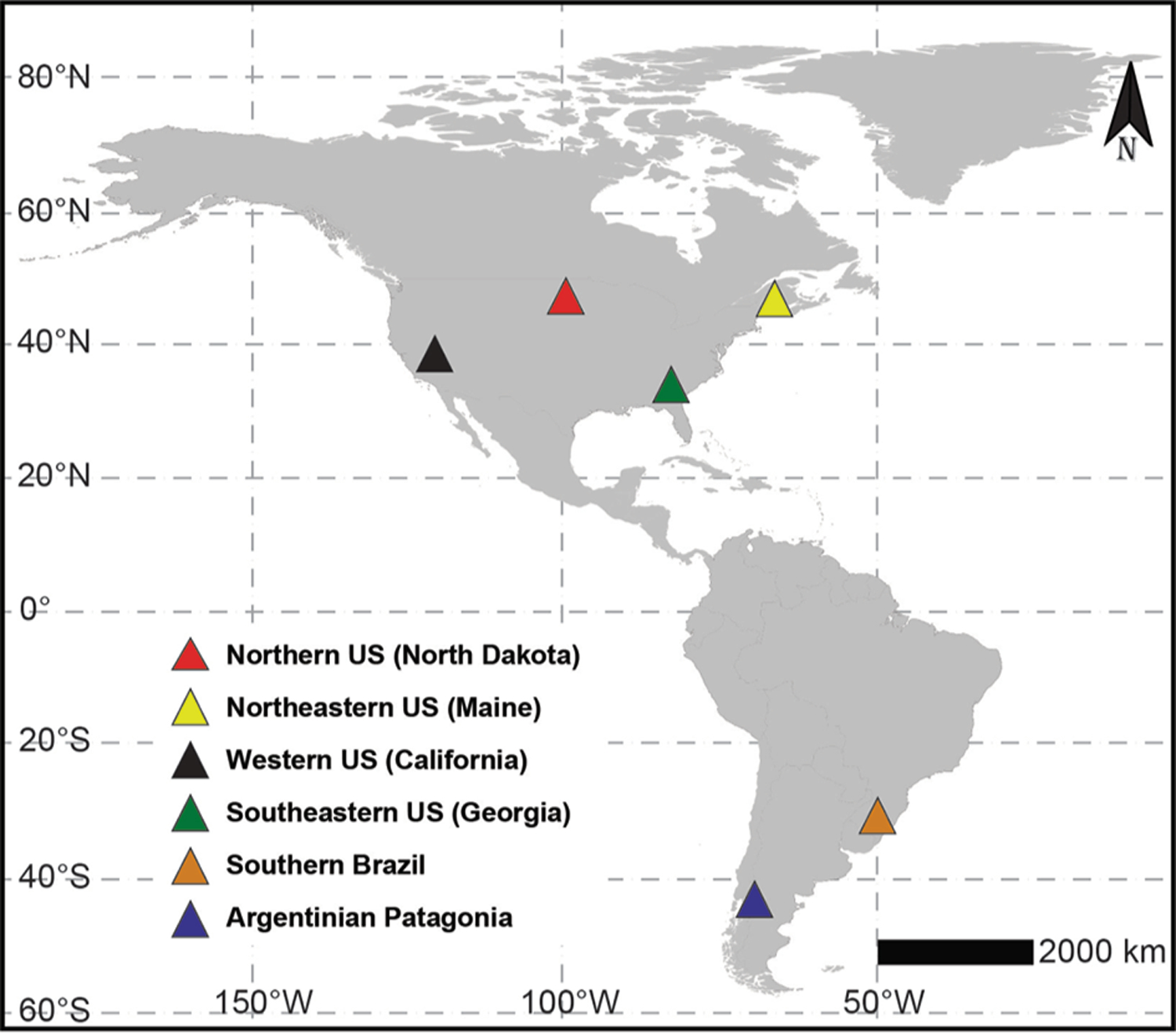
Location (centroid coordinates; filled triangles) of the regions across North and South America.

We compiled data sets of wetland invertebrate assemblages from six regions distributed across North and South America where freshwater wetlands are common elements of the landscape. In North America, the data sets covered the following regions of the continental United States (US): the dry-temperate northern Prairie Pothole region (state of North Dakota), the wet-temperate Northeastern US (state of Maine), dry-temperate Western US (the Central Valley region of the state of California), and the wet-subtropical Southeastern US (Coast Plain of the state of Georgia). In South America, the data sets covered the wet-subtropical Coastal Plain of Southern Brazil (states of Santa Catarina and Rio Grande do Sul) and the dry-temperate Argentinian Patagonia (province of Chubut) (Fig. 1; Table 1).

Each data set was comprised of 10–40 wetlands and the data sets over the six regions included a wide range of habitat types (e.g., prairie potholes, Carolina bays, Patagonian mallines, temporary and permanent ponds, rock pools) and variable time frames (ranging from a single-year snapshot collection to a three-year collection period; Table 1). Thus, our analyses were carried out for each data set separately. Additional details on specific environmental features of the study sites and collection procedures in each study region can be found in Supporting information 1.

### 2.2. Taxonomic and numerical resolution

To compare the effects of taxonomic and numerical resolution on the patterns of community structure in wetland invertebrates, we assembled four matrices: (i) presence-absence at the coarse taxonomic level; (ii) presence-absence at the finest practical taxonomic level; (iii) relative abundance at the coarse taxonomic level; and (iv) relative abundance at the finest practical taxonomic levels. The coarse category usually corresponded to family level (although sometimes certain taxa could only be identified to coarser (higher) taxonomic resolutions, e.g., turbellarians, water mites, some anostracans, etc.). We refer hereafter to this category as the ‘family’ level, for the sake of brevity. The finest practical taxonomic level corresponded to either genus, species or morphospecies; (although certain taxa could only be identified to a coarser taxonomic resolution (e.g. family). We refer hereafter to this resolution as the ‘finest’ level.

### 2.3. Data analysis

#### 2.3.1. Univariate approaches

For each region, we used the Spearman correlation coefficient to test the relationship between ‘family-’ and ‘finest-level’ datasets in terms of richness and community equitability (Shannon-Weiner diversity index). Correlations were conducted separately for each study region.

#### 2.3.2. Multivariate approaches

We assessed whether taxonomic resolution interferes in the homogeneity of multivariate dispersion within each study region. To calculate the average distance of each wetland (sampling unit) to their corresponding group centroid, we employed the PERMDISP procedure (Anderson et al., 2006). In our procedures, beta diversity was given by the total variance in a data set, in accordance with Legendre and De Cáceres (2013); the relative contributions of the turnover and nestedness components were calculated using the Podani family of indices (Podani and Schmera, 2011). We tested for possible effects of taxonomic and numerical resolution on beta diversity metrics and the relative contribution of the turnover (replacement) and nestedness (richness) components to compositional dissimilarity by means of paired t-tests.

We assessed the effects of different resolutions (taxonomic and numerical) on the distribution and relationships between sampling units by means of Principal Coordinates Analysis (PCoA). Prior to the PCoA, the dissimilarity matrices derived from the relative abundance data sets were square-root transformed to avoid the production of negative eigenvalues (Legendre and Legendre, 2012). Finally, we used a Procrustes analysis to test the degree of congruence between PCoA sampling scores derived from datasets with different taxonomic resolutions (Legendre and Legendre, 2012). In our procedures, the Procrustes analysis was based on the site scores of the full set of vectors generated by PCoA. The significance of the Procrustes correlation was assessed with a permutation-based approach (*protest* function; 9999 permutations; Peres-Neto and Jackson, 2001). We tested the degree of congruence for the following set of pairwise combinations of composition data sets: (i) family level (presence-absence vs. relative abundance); (ii) finest level (presence-absence vs. relative abundance); (iii) family level (presence-absence) vs. finest level (presence-absence); and (iv) family level (relative abundance) vs. finest level (relative abundance). We ran all analyses in R v. 3.6.0 (R Core Team, 2019) using the functions available in the packages *vegan* (Oksanen et al., 2019) and *ade4* (Dray and Dufour, 2007).

Finally, to provide a raw assessment of the ratios of the taxa classified to genus or species to their corresponding composite family taxa in each study region, we parsed the character of the “finest level” for each of the six regions, as compiled by the different research groups. That is, we calculated the following information for each data set: (i) the number of taxa that could be identified only to the family level (or to a coarser taxonomic resolution); (ii) the number of families with only one genus; (iii) the number of families with two genera; and (iv) the number of families with three (or more) genera.

## 3. Results

**Fig. 2.**
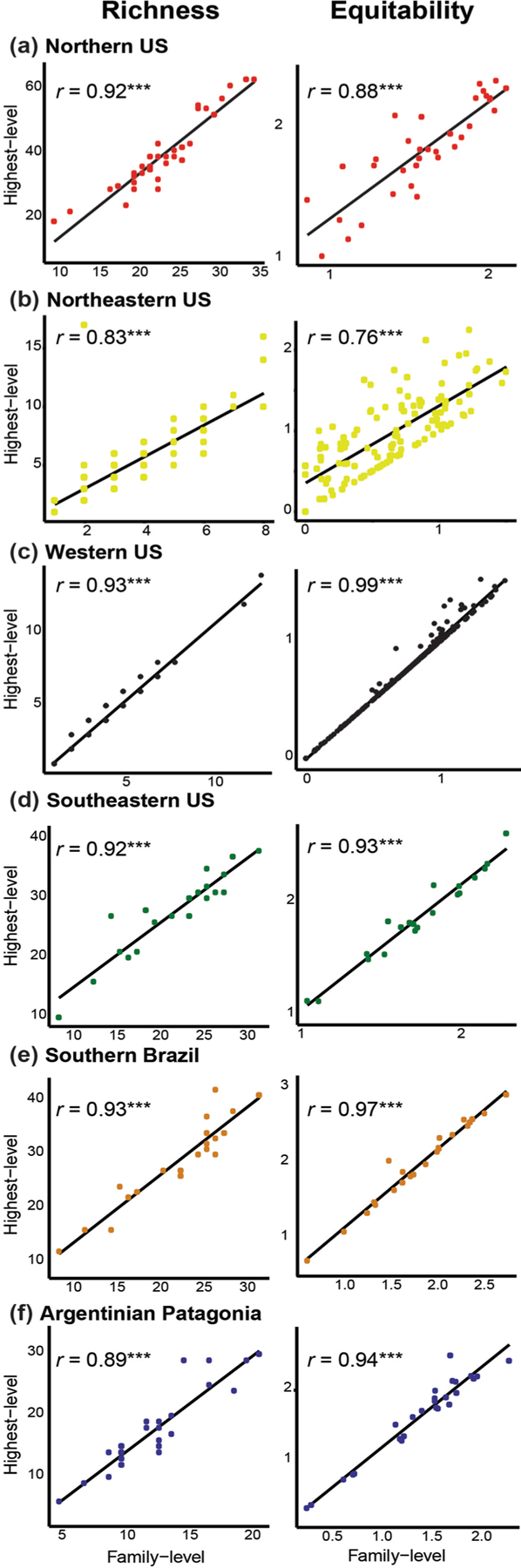
Scatterplots of the relationships between richness (left column) and equitability (right column) of invertebrate communities calculated for each taxonomic resolution in each region. Lines were fitted for the purpose of visualization only.

**Fig. 3.**
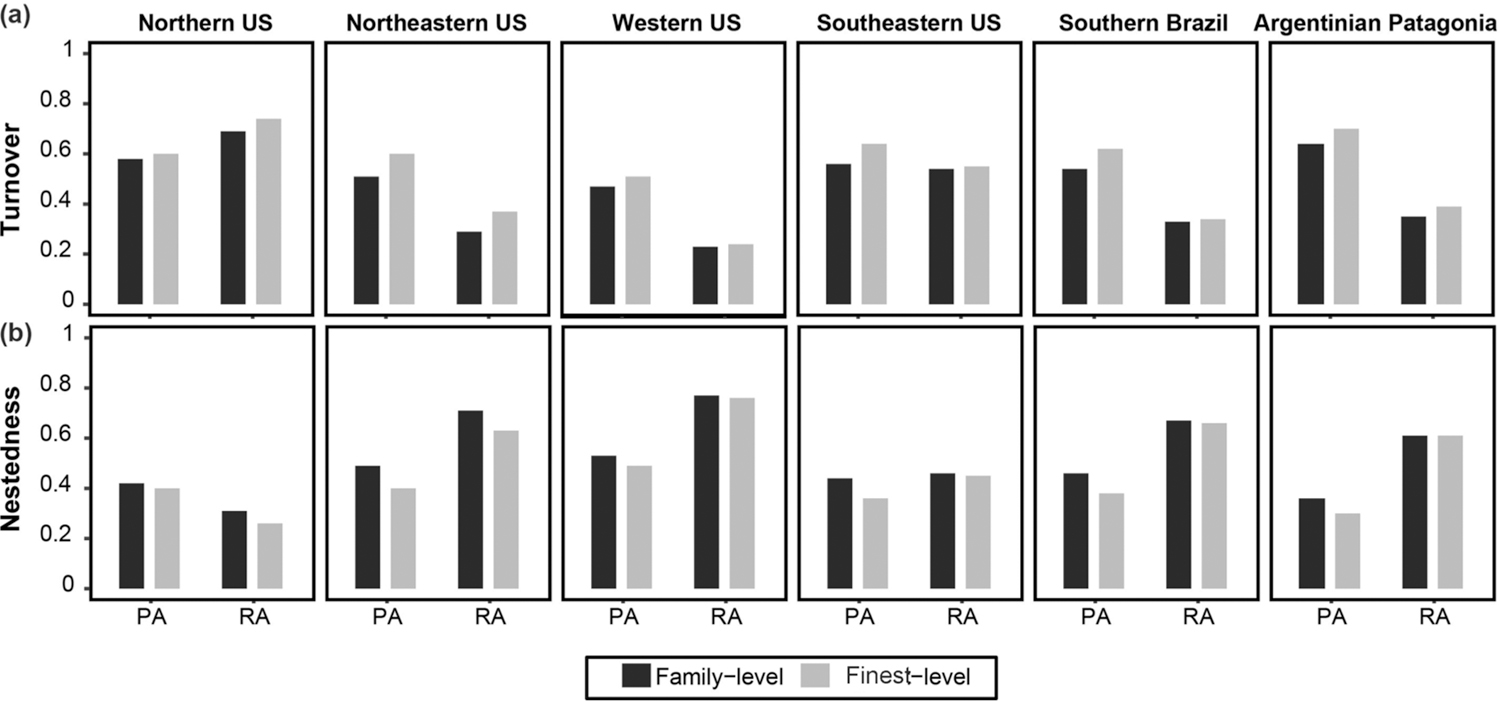
Side-by-side bar charts with the relative contribution of the turnover (a) and nestedness (b) components of the beta diversity across taxonomic and numerical resolutions in each region. PA = presence-absence; RA = relative abundance.

**Fig. 4.**
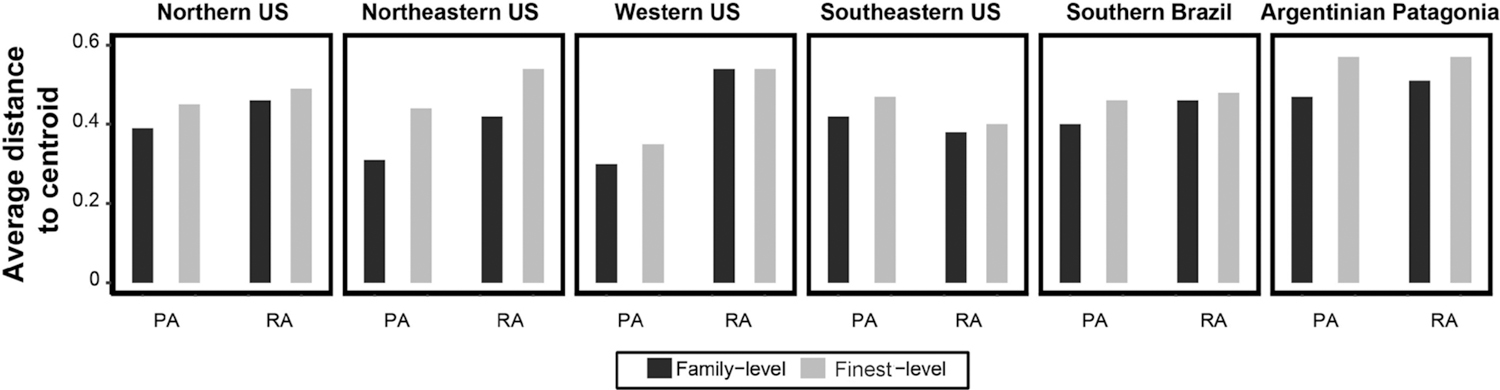
Heterogeneity in community composition depicted in side-by-side bar charts with the average distance to centroid (calculated using the PERMDISP approach) at each taxonomic resolution in each region. PA = presence-absence; RA = relative abundance.

The Spearman correlation coefficients showed that richness values and equitability (measured for each taxonomic resolution) were positively and significantly correlated (*P* < 0.05) in the data sets of all regions (Fig. 2). The numerical outputs of the linear correlations in each region are given in Supporting information 2. For beta diversity, higher values of total beta diversity and turnover were obtained with data with fine (genus) taxonomic resolution than with data with coarse (family) taxonomic resolution; the opposite pattern was found for nestedness component, i.e., lower values of the nestedness component were obtained with data with fine taxonomic resolution as compared to data with coarse taxonomic resolution (irrespective of the numerical resolution; Supporting information 3). Regarding numerical resolution, the relative contribution of the turnover component was higher in presence-absence datasets when compared to relative abundance (except for Northern US data set) (Fig. 3). The outputs of the beta diversity metrics obtained for each numerical and taxonomic resolutions are given in Supporting information 3. Heterogeneity of community composition increased from coarsest to finest taxonomic resolutions and was higher in relative abundance data sets (compared to presence-absence data sets) in each region (Fig. 4). The outputs of the paired t-tests for the comparison of the absolute values and relative contribution of the turnover and nestedness fractions of beta diversity were significant or marginally significant in all cases (Supporting information 4). The Procrustes correlation coefficients for all pairwise comparisons were significant (*P* < 0.0001). Nevertheless, in each region, the values of the Procrustes correlation coefficients were consistently higher (*r* > 0.9 in most cases) for pairwise comparisons between taxonomic resolutions (family vs. finest) than comparisons between numerical resolution (presence-absence vs. relative abundance; Table 2). The graphical outputs of the Procrustes analyses between the invertebrate composition data sets are given in Supporting information 5. PCoA ordination diagrams showed that the relationships among sampling units were consistently more similar across taxonomic resolutions based on the same numerical resolution for all regions (Supporting information 5).

**Fig. 5.**
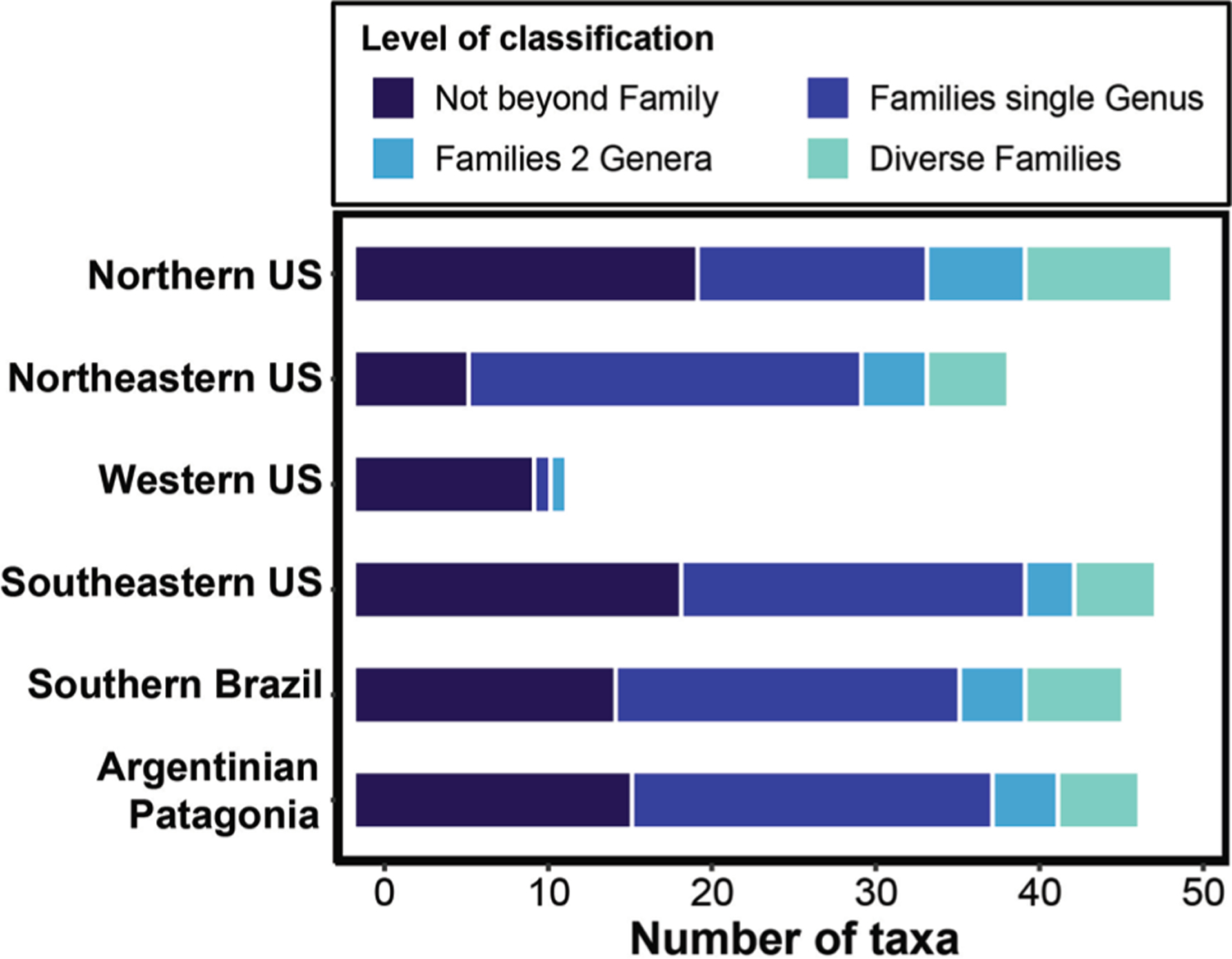
Horizontal stacked bar plots with the number of taxa that could be identified only to the family level (or to a coarser taxonomic resolution) (‘Not beyond Family’); the number of families with only one genus (‘Family Single Genus’); the number of families with two genera (‘Family 2 Genera’); and the number of families with three (or more) genera (‘Diverse Families’) in each of the six study regions.

In every invertebrate composition data set, the number of taxa that could not be identified beyond family levels (or to a coarser taxonomic resolution), combined with the number of families encompassing a single genus, represented the largest amount of the invertebrate composition. In contrast, the number of families where researchers identified multiple genera (or other finer levels) was a distinct minority in each region (Fig. 5).

## 4. Discussion

We found that the patterns observed at coarse taxonomic resolutions (typically family level) showed elevated congruence to finest-practical taxonomic levels (typically genus level) for most of the metrics studied. Most important, the majority of the correlations between taxonomic resolutions were consistent across regions and wetland types. Thus, our results provide empirical evidence for the potential broad application of the higher-taxon approach in studies aiming at the basic characterization of invertebrate community structure patterns in New World freshwater wetlands.

For each alpha diversity metric (richness and Shannon-Weiner index), we found significant and strong correlations between taxonomic resolutions. The majority of the correlations exceeded 0.7, a threshold for congruence suggested by Heino (2010). We found a similar pattern for multivariate metrics, especially the outcomes of ordination techniques (Procrustes tests and PCoA ordination diagrams) of community composition (Table 2; Supporting Information 5). The highest Procrustes *r* coefficients were detected for comparisons between taxonomic (rather than numerical) resolution data sets. Numerous reasons can account for the high congruence between taxonomic resolutions, some of which are related to intrinsic ecological characteristics of wetlands, while others, to contingencies associated with each research group. First, the taxonomic identity in the highest resolution data set often (~80%) matched the identity in the family data set or only a single genus occurred in the same family (Fig. 5). It appears that many families are simply not diverse in wetlands of the Nearctic and Neotropical regions (e.g. Crangonyctidae = *Crangonyx*, Lestidae = *Lestes*, Chaoboridae = *Chaoborus*, etc.); or diverse families frequently encompass a single genus within a given location (Maltchik et al., 2010). Thus, classification differences between family and finest-level occur for a small number of families (0–20% of families had multiple genera; Fig. 5). Low species-to-higher taxa ratios (in our case, finest-to-coarse) underlie the efficacy of the higher-taxon approach (Heino and Soininen, 2007; Bevilacqua et al., 2012; Rosser, 2017, de Oliveira Jr. et al., 2020), and this explanation seems especially prevalent in wetlands.

Besides the low within-family richness, an important reason underlying the high congruence between datasets with different resolutions is the low completeness of the surveys, which, in turn, is due to the lack of expertise by most research groups to classify beyond the family for a wide array of organisms: e.g., helminths, annelids, water mites, microcrustaceans, immature zygopterans and corixids, and certain dipterans. Possibly, if those hard-to-identify groups are also more diverse in wetlands, lower correspondence would be detected between patterns generated with different taxonomic resolutions. Previous studies described that some of these underrepresented taxa can show elevated richness in some regions and be indicative of environmental change (Panatta et al., 2006; Batzer et al., 2014). Alternatively, some of these groups may be so rare that the effort for a detailed taxonomy is deemed unwarranted. Furthermore, because taxonomic errors at the genus level (and lower) are much more likely than at the family level (see Jones, 2008), many researchers opt for a conservative approach. Regrettably the taxonomic expertise needed for a refined taxonomy is often not available, especially for the common invertebrate groups in wetlands, or if available, access to these experts is often beyond the financial capabilities of many research groups, the experts themselves lack the time or resources to do the work, or productive collaborations have not been previously nurtured. Additionally, larval keys for many genus and species almost always focus only on late-instar stages of most invertebrates, which are either underrepresented in samples or unavailable throughout the year, and thus reliable identification even by experts often becomes impracticable. This suggests a scenario in which researchers face the choice of investing more time into finer taxonomic resolutions of fewer speciose groups (in order to obtain more accurate ecological responses) and incurring the risks of increased taxonomic errors along with logistical costs. In summary, low natural richness and limited taxonomic expertise among research groups synergistically act to render similar community-level patterns across different taxonomic resolutions.

To some extent, the differences in taxonomic expertise are likely accountable for the increased beta diversity and average distance to centroid in some datasets, i.e., the Maine (rock pools). In this data set, the Chironomidae were identified to genus (and sometimes species level). This contingency may also explain the much weaker congruence between taxonomic resolutions in the Maine data set. Chironomidae is usually the most speciose (as well as the most abundant and widespread invertebrate group) in freshwater wetlands (e.g., earlier studies have described more than 50 species within a single wetland; see Batzer et al., 2014). In some cases, the species richness of Chironomidae alone can exceed the richness of other invertebrates, vertebrates, or plants. This combination contributes to make the identification of Chironomidae to the genus level likely the most influential contingency impacting the observed community structure patterns (Chessman et al., 2002; King and Richardson, 2002; Jones, 2008). For the highly diverse invertebrate families in wetlands, genus (or species) level resolution has been shown to elucidate finer-level ecological relationships (see King and Richardson (2002) and Chessman et al. (2002) for Chironomidae; Grech et al. (2019) for Culicidae; and McDaniel et al. (2017) for Dytiscidae). Chessman et al. (2002) showed that species-level resolution in Chironomidae data sets rendered more accurate discrimination of sampling units in biomonitoring studies. However, contrary to what we observed in the Maine data set, the classification of the Chironomidae to genus in the Patagonia data set did not have appreciable effects. For organisms other than invertebrates (plants, fishes), the importance of genus (or species) identification for ecological analyses has been highlighted (Mueller et al., 2013). Yet, most wetland research groups are unable to reliably classify Chironomidae specimens beyond sub-family. Although we agree that refined taxonomic identification would always provide more robust assessments, the basic characterization of invertebrate community structure was little affected by the finer-level identification of Chironomidae (and other families) in our study.

The values of beta diversity (total variance in a data set), as well as the relative contribution of the turnover component, and heterogeneity in community composition (average distance to centroid) were higher in the datasets with finest taxonomic resolution. These results resembled the findings by Terlizzi et al. (2009) and Heino (2014), who found lower heterogeneity in multivariate dispersion measures at coarser taxonomic resolutions. To some extent, increased heterogeneity could be the observed outcome of patchy distribution of genera or species, as stressed by Heino (2014). For example, the numerous examples of taxonomic turnover across wetland hydroperiod and predator gradients (Wellborn et al., 1996) typically occur among species within genera (e.g., *Lestes* or *Enallagma* damselflies (Stoks and McPeek, 2006) and *Chaoborus* midges (Garcia and Mittelbach, 2008)) or genera within families (e.g., Limne-philidae caddisflies, Wissinger et al., 2006). This may also be the case in our study, since some of the most widespread wetland invertebrate families (e.g. Dytiscidae, Hydrophilidae, Chironomidae) also encompassed an elevated number of taxa.

The other general trend observed in our assessment were the decreasing strength of the correlations (within the same taxonomic resolutions) and reduced similarity in taxonomic composition ordination diagrams across numerical resolutions. An example of this is evident in the Northern US prairie Pothole and Western US data sets (Supporting Information 5), where the magnitude of correlations decreased more sharply (regardless of taxonomic resolution in both cases). This effect of data transformation was also obtained by Mueller et al. (2013) and Heino (2008) for assorted taxa in streams, with impacts of numerical resolution being especially pronounced for complex community structure analyses (e.g. ordinations). It may be that the choice of changing the numerical resolution masks the effect of the dominant species responsible for driving assemblage patterns in each community (Heino 2008), which is particularly common in wetland invertebrate communities (Kratzer and Batzer, 2007; Batzer et al., 2014; Moraes et al., 2014). Heino (2014) also found that community-environment relationships were more influenced by numerical resolution, regardless of taxonomic resolution. Our results thus suggest that presence-absence resolution may not always be optimal to detect community-environment relationships.

## 5. Conclusion

Our study provides support for the potential application of the higher-taxon approach for the characterization of basic patterns of invertebrate community structure in New World freshwater wetlands. In particular, community-level patterns detected at coarser taxonomic resolutions (typically family level) were similar to those detected with the finest-practical taxonomic levels (usually genus level) for a range of metrics (e.g., richness, equitability and ordination diagrams). Our results thus suggest that family-level assessments may be a cost-effective alternative for biodiversity studies focusing on invertebrate communities. In contrast, the congruence between community composition data sets based on presence-absence and relative abundance resolutions was lower than different numerical resolution, suggesting that the ability to distinguish ecological patterns within study regions is more sensitive to numerical resolution and should be carefully appraised in studies on wetland invertebrates. Lastly, our findings do not render void species-level assessments; these are essential to provide value judgments of wetland habitats for potential legal protection.

## Supplementary Material

1

2

3

4

5

## Figures and Tables

**Table 1:** Basic information about the wetlands in each region used in this study. “Temporal time frame” indicates collection periods.

Continent	Country	Study region (State or Province)	Number of sites	Habitat type	Temporal time frame
North America	United States (US)	Northern US (North Dakota)	17	Prairie potholes: seasonal and permanent	Two years
		Northeastern US (Maine)	40	Rock pools	One year
		Western US (California)	13	Rock pools	Three years
		Southeastern US (Georgia)	10	Carolina bays: seasonal and permanent	Two years
South America	Brazil	Southern Brazil (Rio Grande do Sul) (Santa Catarina)	12	Temporary ponds	Two years
	Argentina	Patagonia (Chubut)	26	Patagonian mallines: seasonal and permanent	One year

**Table 2:** Results of the Procrustes analysis between the invertebrate composition data sets with different taxonomic and numerical resolutions. PA = presence-absence; RA = relative abundance; ‘Family’ = family-level taxonomic resolution; ‘Finest’ = finest practical taxonomic level.

Study region	Northern US		Northeastern US		Western US		Southeastern US		Southern Brazil		Argentinian Patagonia	
Procrustes contrasts	*r*	*P*	*r*	*P*	*r*	*P*	*r*	*P*	*r*	*P*	*r*	*P*
Family - PA vs. Family - RA	0.798	0.0001	0.61	0.0001	0.561	0.0001	0.835	0.0001	0.867	0.0001	0.885	0.0001
Finest - PA vs. Finest - RA	0.88	0.0001	0.747	0.0001	0.567	0.0001	0.876	0.0001	0.897	0.0001	0.932	0.0001
Family - PA vs. Finest - PA	0.945	0.0001	0.809	0.0001	0.921	0.0001	0.979	0.0001	0.968	0.0001	0.94	0.0001
Family - RA vs. Finest - RA	0.972	0.0001	0.829	0.0001	0.978	0.0001	0.996	0.0001	0.998	0.0001	0.98	0.0001

## Data Availability

Federally funded data generated during this study are available from the USGS at https://www.sciencebase.gov/catalog/item/599d9555e4b012c075b964a6 (Mushet et al., 2017).

## References

[R1] Anderson MJ, Ellingsen KE, McArdle BH, 2006. Multivariate dispersion as a measure of beta diversity. Ecol. Lett 9, 683–693. 16706913 10.1111/j.1461-0248.2006.00926.x

[R2] Anderson MJ, Crist TO, Chase JM, Vellend M, Inouye BD, 2011. Navigating the multiple meanings of beta diversity: a roadmap for the practicing ecologist. Ecol. Lett 14, 19–28. 21070562 10.1111/j.1461-0248.2010.01552.x

[R3] Bailey RC, Norris RC, Reynoldson TB, 2001. Taxonomic resolution of benthic macroinvertebrate communities in bioassessments. J. N. Am. Benthol. Soc 20, 280–286.

[R4] Batzer DP, Cooper R, Wissinger SA, 2014. Wetland animal ecology. In: Batzer DP, Sharitz R (Eds.), Ecology of freshwater and estuarine wetlands University of California Press, Berkeley, pp. 151–184.

[R5] Batzer DP, 2013. The seemingly intractable ecological responses of invertebrates in North American wetlands: a review. Wetlands 33, 1–15.

[R6] Bertrand Y, Pleijel F, Rouse GW, 2006. Taxonomic surrogacy in biodiversity assessments, and the meaning of Linnaean ranks. Syst. Biodivers 4, 149–159.

[R7] Bevilacqua S, Terlizzi A, Claudet J, Fraschetti S, Boero F, 2012. Taxonomic relatedness does not matter for species surrogacy in the assessment of community responses to environmental drivers. J. Appl. Ecol 49, 357–366. 10.1111/j.1365-2664.2011.02096.x. In this issue.

[R8] Chadd R, Extence C, 2004. The conservation of freshwater macroinvertebrate populations: a community-based classification scheme. Aquatic Conserv: Mar. Freshw. Ecosyst 14, 597–624.

[R9] Chessman BC, Trayler KM, Davis JA, 2002. Family- and species-level biotic indices for macroinvertebrates of wetlands on the Swan Coastal Plain. Western Australia. Mar. Freshwater Res 53, 919–930.

[R10] Costanza R, de Groot R, Sutton P, van der Ploeg S, Anderson SJ, Kubiszewski I, Farber S, Turner RK, 2014. Changes in the global value of ecosystem services. Global Environ. Chan 26, 152–158.

[R11] de Oliveira SS, Ortega JCG, dos Santos Ribas LG, Lopes VG, Bini LM, 2020. Higher taxa are sufficient to represent biodiversity patterns. Ecol. Indic 111, 105994.

[R12] Dray S, Dufour A, 2007. The ade4 package: implementing the duality diagram for ecologists. J. Stat. Soft 22, 1–20.

[R13] Epele LB, Miserendino ML, 2015. Environmental quality and aquatic invertebrate metrics relationships at patagonian wetlands subjected to livestock grazing pressures. PLoS ONE 10, e0137873. 26448652 10.1371/journal.pone.0137873 PMC4598092

[R14] Garcia EA, Mittelbach GG, 2008. Regional coexistence and local dominance in Chaoborus: species sorting along a predation gradient. Ecology 89, 1703–1713. 18589534 10.1890/07-0737.1

[R15] Garrido J, Munilla I, 2008. Aquatic Coleoptera and Hemiptera assemblages in three coastal lagoons of the NW Iberian Peninsula: assessment of conservation value and response to environmental factors. Aquatic Conserv: Mar. Freshw. Ecosyst 18, 557–569.

[R16] Grech MG, Manzo LM, Epele LB, Laurito M, Claverie AÑ, Ludueña-Almeida FF, Miserendino ML, Almirón WR, 2019. Mosquito (Diptera: Culicidae) larval ecology in natural habitats in the cold temperate Patagonia region of Argentina. Parasites Vectors 12, 1–14. 31064397 10.1186/s13071-019-3459-y PMC6505294

[R17] Heino J, 2008. Influence of taxonomic resolution and data transformation on biotic matrix concordance and assemblage-environment relationships in stream macroinvertebrates. Boreal Environ. Res 13, 359–369.

[R18] Heino J, 2010. Are indicator groups and cross-taxon congruence useful for predicting biodiversity in aquatic ecosystems? Ecol. Indic 10, 112–117.

[R19] Heino J, 2014. Taxonomic surrogacy, numerical resolution and responses of stream macroinvertebrate communities to ecological gradients: are the inferences transferable among regions? Ecol. Indic 36, 186–194.

[R20] Heino J, Soininen J, 2007. Are higher taxa adequate surrogates for species-level assemblage patterns and species richness in stream organisms? Biol. Cons 137, 78–89.

[R21] Hernandez FJ, Carassou L, Graham WM, Powers SP, 2013. Evaluation of the taxonomic sufficiency approach for ichthyoplankton community analysis. Mar. Ecol. Prog. Ser 491, 77–90.

[R22] Hortal J, de Bello F, Diniz-Filho JAF, Lewinsohn TM, Lobo JM, Ladle RJ, 2015. Seven shortfalls that beset large-scale knowledge of biodiversity. Annu. Rev. Ecol. Evol. Syst 46, 523–549.

[R23] Jones FC, 2008. Taxonomic sufficiency: the influence of taxonomic resolution on freshwater bioassessments using benthic macroinvertebrates. Environ. Rev 16, 45–69.

[R24] Kallimanis AS, Mazaris AD, Tsakanikas D, Dimopoulos P, Pantis JD, Sgardelis SP, 2012. Efficient biodiversity monitoring: which taxonomic level to study. Ecol. Indic 15, 100–104.

[R25] King RS, Richardson CJ, 2002. Evaluating subsampling approaches and macroinvertebrate taxonomic resolution for wetland bioassessment. J. N. Am. Benthol. Soc 2002 (21), 150–171.

[R26] Kratzer EB, Batzer DP, 2007. Spatial and temporal variation in aquatic macroinvertebrates in the Okefenokee Swamp, Georgia, USA. Wetlands 27, 127–140.

[R27] Legendre P, Legendre LFJ, 2012. Numerical Ecology, third ed. Elsevier, Amsterdam.

[R28] Legendre P, De Cáceres M, 2013. Beta diversity as the variance of community data: dissimilarity coefficients and partitioning. Ecol. Lett 16, 951–963. 23809147 10.1111/ele.12141

[R29] Lenat DR, Resh VH, 2001. Taxonomy and stream ecology – the benefits of genus and species-level identifications. J. N. Am. Benthol. Soc 20, 287–298.

[R30] Maltchik L, Stenert C, Spies MR, Siegloch AE, 2010. Diversity and distribution of Ephemeroptera and Trichoptera in Southern Brazil Wetlands. J. Kansas Entomol. Soc 82, 160–173.

[R31] McDaniel CH, McHugh JV, Batzer DP, 2017. Congeneric predaceous diving beetle species fail to segregate in a floodplain system: a case of amplified sympatry. Environ. Entomol 46, 494–501. 28430893 10.1093/ee/nvx063

[R32] Melo AS, 2005. Effects of taxonomic and numeric resolution on the ability to detect ecological patterns at a local scale using stream macroinvertebrates. Arch. Hydrobiol 164, 309–323.

[R33] Monk WA, Wood PJ, Hannah DM, Extence CA, Chadd RP, Dunbar MJ, 2012. How does macroinvertebrate taxonomic resolution influence ecohydrological relationships in riverine ecosystems. Ecohydrology 5, 36–45.

[R34] Moraes AB, Stenert C, Rolon AS, Leonardo Maltchik L, 2014. Effects of landscape factors and hydroperiod on aquatic macroinvertebrates with different dispersal strategies in southern Brazil ponds. J. Freshwater Ecol 29, 319–355.

[R35] Mueller M, Pander J, Geist J, 2013. Taxonomic sufficiency in freshwater ecosystems: effects of taxonomic resolution, functional traits, and data transformation. Freshw. Sci 32, 762–778.

[R36] Mushet DM, Euliss NH Jr., and Solensky MJ 2017, Cottonwood Lake Study Area - Invertebrate Counts, U.S. Geological Survey data release, 10.5066/F7BK1B77.

[R37] Neeson TM, van Rijn I, Mandelik Y, 2013. How taxonomic diversity, community structure, and sample size determine the reliability of higher taxon surrogates. Ecol. Appl 23, 1216–1225. 23967587 10.1890/12-1167.1

[R38] Oksanen J, Blanchet FG, Friendly M, Kindt R, Legendre P, McGlinn D, Minchin PR, O’Hara RB, Simpson GL, Solymos P, Stevens MHM, Szoecs E, Wagner H, 2019. vegan: Community Ecology Package. R package version 2.5–6

[R39] Panatta A, Stenert C, Freitas SMF, Maltchik L, 2006. Diversity of chironomid larvae in palustrine wetlands of the coastal plain in the south of Brazil. Limnology 7, 23–30.

[R40] Peres-Neto PR, Jackson DA, 2001. How well do multivariate data sets match? The advantages of a Procrustean superimposition approach over the Mantel tests. Oecologia 129, 169–178. 28547594 10.1007/s004420100720

[R41] Podani J, Schmera D, 2011. A new conceptual and methodological framework for exploring and explaining pattern in presence-absence data. Oikos 120, 1625–1638.

[R42] R Core Team, 2019. R: A language and environment for statistical computing R Foundation for Statistical Computing, Vienna, Austria. URL https://www.Rproject.org/.

[R43] Rosser N, 2017. Shortcuts in biodiversity research: what determines the performance of higher taxa as surrogates for species? Ecol. Evol 7, 2595–2603. 28428850 10.1002/ece3.2736 PMC5395451

[R44] Rosser N, Eggleton P, 2011. Can higher taxa be used as a surrogate for species-level data in biodiversity surveys of litter/soil insects? J. Insect Conserv 16, 87–92.

[R45] Sgarbi LF, Bini LM, Heino J, Jyrkänkallio-mikkola J, Landeiro VL, Santos EP, Melo AS, 2020. Sampling effort and information quality provided by rare and common species in estimating assemblage structure. Ecol. Indicat 110, 105937.

[R46] Simić V, Simić S, Paunović M, Cakić P, 2007. Model of the assessment of the critical risk of extinction and the priorities of protection of endangered aquatic species at the national level. Biodivers. Conserv 16, 2471–2493.

[R47] Stoks R, McPeek MA, 2006. A tale of two diversifications: Reciprocal habitat shifts to fill ecological space along the pond permanence gradient. Am. Nat 168, 50–72. 10.1086/509045 17109329

[R48] Terlizzi A, Anderson MJ, Bevilacqua S, Fraschetti S, Wlodarska-Kowalczuk M, Ellingsen KE, 2009. Beta diversity and taxonomic sufficiency: do higher level taxa reflect heterogeneity in species composition? Divers. Distrib 15, 450–458.

[R49] Vilmi A, Karjalainen SM, Nokela T, Tolonen K, Heino J, 2016. Unravelling the drivers of aquatic communities using disparateorganismal groups and different taxonomic levels. Ecol. Indic 60, 108–118.

[R50] Wellborn GA, Skelly DK, Werner EE, 1996. Mechanisms creating community structure across a freshwater habitat gradient. Annu. Rev. Ecol. S 27, 337–364.

[R51] Wheeler QD, Raven PH, Wilson EO, 2004. Taxonomy: impediment or expedient? Science 303, 285. 14726557 10.1126/science.303.5656.285

[R52] Wissinger SA, Whissel JC, Eldermire C, Brown WS, 2006. Predator defense along a permanence gradient: roles of case structure, behavior, and developmental phenology in caddisflies. Oecologia 147, 667–678. 16463178 10.1007/s00442-005-0303-1

