# Supplementary material for: Does taxonomic and numerical resolution affect the assessment of invertebrate community structure in New World freshwater wetlands?
Source: Ecol Indic. Author manuscript; Available in PMC 2021 Jun 1. (PMC7963273; doi:10.1016/j.ecolind.2021.107437)
Supplement: 1 [file NIHMS1668784-supplement-1.doc]

# Pires et al. 2020. Does taxonomic and numerical resolution affect the assessment of invertebrate community structure in New World freshwater wetlands? Ecol. Indicat. submitted. Supporting Information 1. Detailed descriptions of the environmental characterization, field sampling procedures and laboratory methods used in each region.

[1. Northern US (state of North Dakota) 2](#__RefHeading___Toc48638447)

[Study region 2](#__RefHeading___Toc48638448)

[Sampling design 2](#__RefHeading___Toc48638449)

[Field methods 2](#__RefHeading___Toc48638450)

[Laboratory procedures 3](#__RefHeading___Toc48638451)

[References 3](#__RefHeading___Toc48638452)

[2. Northeastern US (state of Maine) *(modified from Gagne 2019)* 4](#__RefHeading___Toc48638453)

[Study region 4](#__RefHeading___Toc48638454)

[Sampling design 4](#__RefHeading___Toc48638455)

[Field methods 5](#__RefHeading___Toc48638456)

[Laboratory procedures 5](#__RefHeading___Toc48638457)

[References 5](#__RefHeading___Toc48638458)

[3. Western US (state of California) 6](#__RefHeading___Toc48638459)

[Study region 6](#__RefHeading___Toc48638460)

[Sampling design 6](#__RefHeading___Toc48638461)

[Field methods 6](#__RefHeading___Toc48638462)

[References 6](#__RefHeading___Toc48638463)

[4. Southeastern US (state of Georgia) 8](#__RefHeading___Toc48638464)

[Study region 8](#__RefHeading___Toc48638465)

[Sampling design and field methods 9](#__RefHeading___Toc48638466)

[Laboratory procedures 9](#__RefHeading___Toc48638467)

[References 9](#__RefHeading___Toc48638468)

[5. Southern Brazil (states of Rio Grande do Sul and Santa Catarina) 10](#__RefHeading___Toc48638469)

[Study region 10](#__RefHeading___Toc48638470)

[Sampling design 10](#__RefHeading___Toc48638471)

[Field methods and laboratory procedures 10](#__RefHeading___Toc48638472)

[References 11](#__RefHeading___Toc48638473)

[6. Argentinean Patagonia (province of Chubut) *(modified from Epele et al. 2019)* 12](#__RefHeading___Toc48638474)

[Study region 12](#__RefHeading___Toc48638475)

[Invertebrate collection and laboratory procedures 12](#__RefHeading___Toc48638476)**References 12**

# [1. Northern US (state of North Dakota)](#__RefHeading___Toc48638477)

## Study region

The Prairie Pothole Region is a wetland-grassland dominated landscape (~777,000 km^2^) extending from northwest Iowa, North and South Dakota and into central Alberta (Smith et al. 1964). The region is characterized by millions of individual wetland basins that can exhibit highly variable ponded-water dynamics (Gleason et al. 2011). The high spatial and temporal variability exhibited by prairie-pothole wetland ponds is a reflection of the high spatial and temporal variability in climate (Winter 2003; Liu & Schwartz 2012). Annual maximum and minimum temperatures can reach 40 °C in the summer and -40 °C in the winter, respectively, and mean annual precipitation ranges from 30 cm/year to 90 cm/year. The US portion of the Prairie-Pothole Region is typically warmer and wetter than the Canadian portion.

## Sampling design

Seventeen depressional prairie-pothole wetlands collectively known as the Cottonwood Lake Study Area (hereafter CLSA) were selected for this study using data from the 2013 and 2014 sampling seasons. The CLSA is part 92-ha complex of natural prairie-embedded wetlands located on a U.S Fish and Wildlife Service managed Waterfowl Production Area in Stutsman County (ND). The sampled wetlands occur along ponded-water permanence gradients, whereas of the 17 wetlands monitored, eight of the wetlands are seasonally ponded and nine wetlands as permanently ponded (McLean et al. 2019). When ponded, wetland surface-water areas for the sampled wetlands ranged from 20 to 232,602 m^2^, during the 2013 to 2014 sampling seasons.

## Field methods

Aquatic macroinvertebrates were sampled during the ice-free growing months (April to September) in 2013 and 2014 using vertically oriented, funnel traps (Swanson, 1978) and deployed in wetlands for 24 h. Samples were collected once a month from random locations within each vegetation zone present (shallow marsh, deep marsh, open water) along three transects radiating from the center of each wetland. Traps are only placed in areas of the wetlands with surface water depth > 25 cm. A maximum of 54 samples are collected from a wetland a year if all three vegetation zones contain ponded water for all six sampling months.

## Laboratory procedures

Upon collection, trap contents are sieved through a 0.5-mm screen into a 475-ml plastic sample container, preserved with ethyl alcohol (80%) and stored in a U.S. Geological Survey laboratory in Jamestown (ND), until processed. Processing consists of rinsing samples through a stainless-steel beaker with a 0.5-mm screened side and then separating remaining aquatic macroinvertebrates (> 0.5 mm) from debris and identifying them to the lowest attainable taxonomic resolution (typically to genus, Mushet et al. 2017). Aquatic-macroinvertebrate sampling data for all seventeen wetlands was then summarized as mean-annual abundances of uniquely identified taxa per trap. Wetland macroinvertebrate-monitoring data from the CLSA are openly available from the U.S. Geological Survey through the Missouri Coteau Wetland Ecosystem Observatory: <https://www.sciencebase.gov/catalog/item/52f0ffd9e4b0f941aa181fc6>.

## References

Gleason, R.A., Euliss Jr, N.H., Tangen, B.A., Laubhan, M.K., Browne, B.A. 2011. USDA conservation program and practice effects on wetland ecosystem services in the Prairie Pothole Region. Ecol. Appl. 21, 65–81.

Liu, G., Schwartz, F.W. 2011. An integrated observational and model-based analysis of the hydrologic response of prairie pothole systems to variability in climate. Water Resour. Res. 47, W02504.

McLean, K.I., Mushet, D.M., Sweetman, J.N., Anteau, M.J., Wiltermuth, M.T. 2019. Invertebrate communities of Prairie-Pothole wetlands in the age of the aquatic Homogenocene. Hydrobiologia. <https://doi.org/10.1007/s10750-019-04154-4>.

Mushet, D.M., Euliss Jr., N.H., Solensky, M.J. 2017. Cottonwood Lake Study Area- Invertebrate Counts. U.S. Geological Survey Data Release. <https://doi.org/10.5066/F7BK1B77>.

Smith, A.G., Stoudt, J.H., Gollop, J.B. 1964. Prairie potholes and marshes, in: Linduska, J.P. (Ed.), Waterfowl tomorrow. U.S. Government Printing Office. Washington, DC, pp. 39–50.

Swanson, G.A. 1978. Funnel trap for collecting littoral aquatic invertebrates. Prog. Fish. Cult. 40, e73.

Winter, T.C. 2003. Hydrological, chemical, and biological characteristics of a prairie pothole wetland complex under highly variable climate conditions – The Cottonwood Lake Area, east-central North Dakota. USGS Professional Paper 1675, 109 pp.

# 2. Northeastern US (state of Maine) *(modified from Gagne 2019)*

## Study region

Four exposed rock outcrops were selected as sites along the Penobscot River between Old Town and Eddington, Maine. Sites were designated a number based on location along the river, with Site 1 being the furthest upstream and Site 4 being the furthest downstream. Site 1 was the least likely to be flooded by the river during the summer (based on vertical distance from the river in June), whereas Sites 3, 4, and parts of Site 2 were prone to regular flooding events. All sampled areas except for an elevated bench of Site 2 were flooded by the river during peak spring runoff in April. Site 3 was the only site that was located directly underneath riparian trees, whereas the other three sites were well-removed (> 15 m) from the riparian zone (Fig. 1).


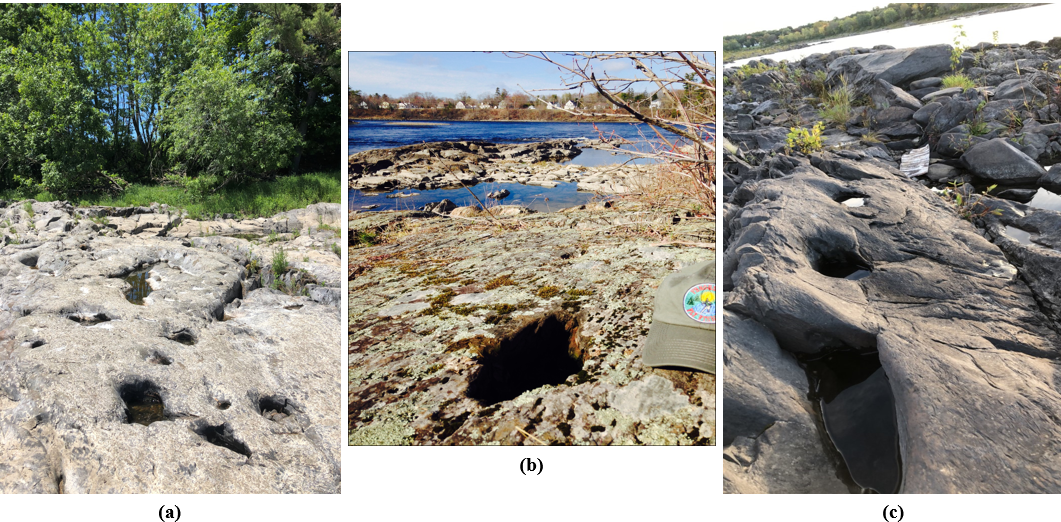


**Fig. 1.** Examples of rock outcrops and pools sampled. Site 3 (a), Site 2 from the pool closest to the riparian zone (b), and Site 4 (c) along the Penobscot River near Milford, Maine.

## Sampling design

Ten rock pools between 10 cm and 1 m in diameter were sampled at each site between June 20 and September 26, 2018. Pools were chosen in a stratified manner throughout each site to account for different microhabitats in different areas of the outcrops. Pool elevation with respect to the adjacent river varied within sites, such that during an increase in river height one or several pools were likely to be flooded with river water. Pool volumes ranged from 0.72 to 28.05 L, with a median of 4.07 L.

## Field methods

The aquatic invertebrate communities were sampled in each pool in late June, early August, and mid-September. Invertebrates were collected with a fine-mesh (250 µm) aquarium net by sweeping through the pool for three minutes after the substrate had been disturbed. Samples were immediately transferred to 70% ethyl alcohol and stored for later sorting and identification.

## Laboratory procedures

All invertebrates, with the exception of mosquitos, zooplankton, bivalves, and gastropods, were identified to genus using Peckarsky et al. (1990) and Merritt et al. (2008). The latter three taxa were identified only to family and mosquitos were identified to species using Andreadis et al. (2005). Chironomids were subsampled by first randomly selecting twelve individuals from each sample to mount, then visually scanning the remaining sample and picking out unique individuals that were missed. Chironomid larvae were slide-mounted in CMC-10 following methods outlined by Epler (2001).

## References

Andreadis, T.G., Thomas, M.C., Shepard, J.J. 2005. Identification guide to mosquitoes of Connecticut. Experiment Station Bulletin 966. The Connecticut Agricultural Experiment Station, New Haven, CT.

Epler, J.H. 2001. Identification manual for the larval Chironomidae (Diptera) of North and South Carolina. North Carolina Dep. Envir. Natural Resources, Div. Water Quality.

Gagne, C. 2019. Spatial and temporal variation in the aquatic invertebrate community structure of rock pools along the Penobscot River, Maine. MS thesis, School of Biology and Ecology, University of Maine, Orono, Maine.

Merritt, R., Cummins, K., Berg, M.B. 2008. An Introduction to the Aquatic Insects of North America, fifth ed. Kendall/Hunt, Dubuque, Iowa.

Pecarsky, B.L., Fraissinet, P.R., Penton, M.A., Conklin, D.J. 1990. Freshwater macroinvertebrates of northeastern North America. Cornell University Press, Ithaca.

# 3. Western US (state of California)

## Study region

Rock pools (Fig. 2) were sampled from eight sites within a ~25 km^2^ region in Contra Costa and Alameda Counties. The Mediterranean climate of this region is characterized by cool wet winters and hot and dry summer and falls. The hydrological cycle is highly variable across years because of the highly variable precipitation patterns. Nonetheless, because of the impermeable substrate, the first consistent rains lead to inundation, usually by November, and desiccate by May (Marr 2019). Each site had at least one sandstone rock outcrop with multiple pools on each outcrop.

## Sampling design

Rock pools were sampled between November and April during the three years (2016-2019), as part of a monitoring program of the endangered species *Branchinecta longiantenna* (Eng et al. 1990). Pools containing *B*. *longiantenna* were targeted along with 2-4 adjacent pools. During the first year 97 pools were sampled, 57 during the second year, and 87 during the third year. Pools are highly variable in size (depth (cm): mean = 12.4, SD = 9.5).

## Field methods

Large aquarium net (20.3 x 15.2 cm, 0.2 mm mesh) was used for a 10-s sweep capturing the depth and topography of the pool. Individuals were placed into a tray for identification and enumeration. Individuals are placed into a tray for enumeration of individuals of each taxa (see form above). Voucher specimens were collected for further identification, using Thorp & Covich (2010). Observations (~1 min) of the pool are made for rare taxa not caught to be recorded.

## References

Eng, L.L., Belk, D., Eriksen, C.H. 1990. Californian Anostraca: distribution, habitat, and status. J. Crustacean Biol. 10, 247–277.

Marr, K.D., 2019. Hydrology, environment, and community structure associated with the seasonal occurrence of *Branchinecta lynchi* in rock pools. M.S. Thesis, Department of Biological Sciences, California State University, Sacramento, CA.

Thorp, J.H., Covich, A.P. 2010. Ecology and classification of North American freshwater invertebrates. 3rd edition. Academic Press, New York.


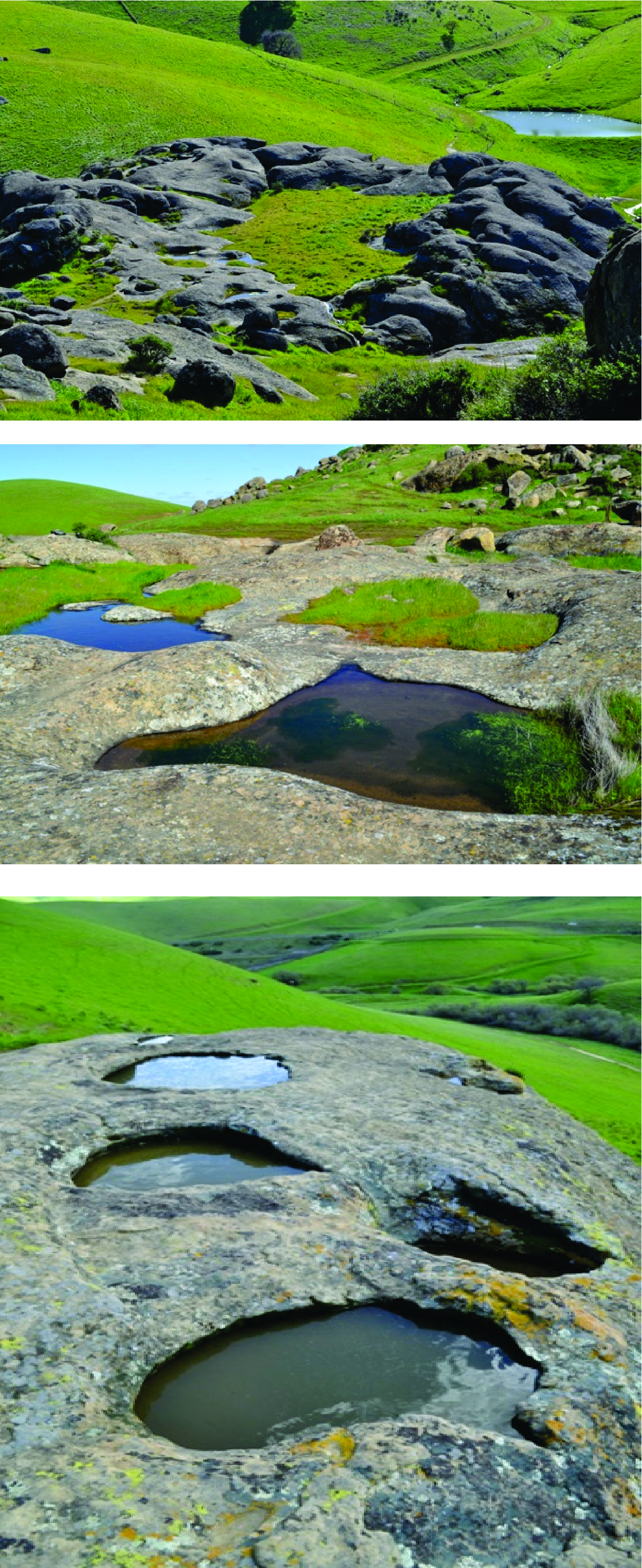


**Fig. 2.** Examples of rock pools and outcrops that were sampled in Contra Costa and Alameda Counties (Central Valley region of the state of California).

# 4. Southeastern US (state of Georgia)

## Study region

Ten Carolina bay wetlands were selected for study, located on Georgia’s Tuckahoe Wildlife Management Area (Screven County). Carolina bays are depression wetlands, common on the upper Atlantic Coastal Plain of the Southeastern US (Kirkman et al. 2012). Water budgets are dominated by inputs of rainfall and outputs of evapotranspiration; groundwater discharge into wetlands rarely occurs. The 10 study wetlands had near natural hydrologies, and waters were oligotrophic (pH 3.5-5.0; electrical conductivity <100µS/sec). Wetland vegetation was primarily forested (Fig. 3), although emergent and submersed vegetation was common; surrounding uplands were natural pine-hardwood forests. The sites were selected to cover the range of sizes (0.1-100 ha), and hydrologies (seasonal-permanent) that occurred naturally in the area. The local climate is humid, subtropical with hot summers and cool winters, with approximately 100 mm of rainfall occurring most months. Due to increased evapotranspiration in the summer and early fall, seasonally flooded habitats tended to dry then.

## Sampling design and field methods

Each of the 10 Carolina bays was sampled seasonally (March, July, November) from 2015 through 2019. For purposes of this study, only data from 2015 and 2019 were included; they were among the wettest and the driest study years, and thus the full range of hydrological impacts on biota was included in the data set. Samples were collected with a standardized sweep netting procedure (mesh size = 500 µm) that proportionally targeted all sub-habitats that occurred. Samples were not collected from completely dry habitats.

## Laboratory procedures

Field preserved samples (95% ethanol) were transferred to the laboratory for processing. Samples were rinsed in stacked sieves (1-mm and 500-µm), to split the organisms into macro- and micro-invertebrate components (small early stage macroinvertebrates that washed through the 1-mm sieve were recombined with the larger individuals when quantified). Macroinvertebrate portions were randomly split into halves, and one half was hand-picked using a stereomicroscope; if < 100 individuals was recovered from this subsampling, the entire sample was then picked. Microinvertebrate portions were randomly split so that 1/16 (or on occasion 1/8) of the sample was picked. Taxa were identified using keys in Merritt et al. (2008) and Thorp and Covich (2010), to levels where the research team was confident of reliability, and then quantified. A few key taxa names were confirmed by taxonomists at the Georgia Museum of Natural History.

## References

Kirkman, L.K., Smith, L.L., Golladay, S.W., 2012. Southeastern depressional wetlands, in: Batzer, D.P., Baldwin, A.H. (Eds.), Wetland Habitats of North America; Ecology and Conservation Concerns. University of California Press, Berkeley, pp. 203–215.

Merritt, R., Cummins, K., Berg, M.B. 2008. An Introduction to the Aquatic Insects of North America, fifth ed. Kendall/Hunt, Dubuque, Iowa.

Thorp, J.H., Covich, A.P. 2010. Ecology and classification of North American freshwater invertebrates. 3rd ed. Academic Press, New York.


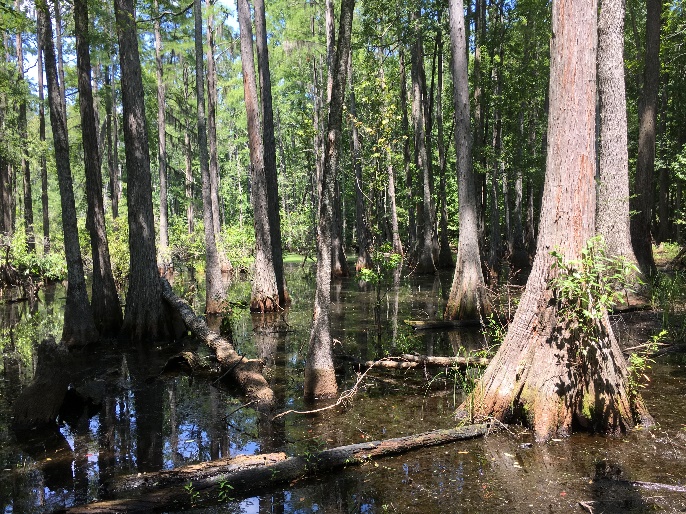


**Fig. 3.** Example of a Carolina bay wetland.

# 5. Southern Brazil (states of Rio Grande do Sul and Santa Catarina)

## Study region

The southern Brazilian Coastal Plain (‘*Planície Costeira*’) runs along ~640-km (and ~80-km wide) the coasts of the states of Santa Catarina (SC) and Rio Grande do Sul (RS). The region is characterized by lowlands (altitudes below < 20 m), sandy geomorphology (Villwock & Tomazelli 2006) and harbors a high number of wetland ecosystems (Maltchik et al. 2003). The climate in the region is type Cfa (humid subtropical without a dry season, according to Köppen’s classification system); the annual average temperature ranges from 12 to 22 ° C, and the annual rainfall, from 1,000 to 1,500 mm (Alvares et al. 2013).

## Sampling design

Twelve seasonal wetlands were selected for sampling along a 520-km latitudinal range in the southern Brazilian Coastal Plain (latitudes 27–31 ° S). We selected the wetlands according to the following criteria: natural condition (i.e., man-made ponds were not selected for sampling), surrounding land use characterized by low anthropic impact (preference was given to wetlands located in non-urbanized areas; the large majority of them were located in areas with non-intensive livestock). The wetlands had a maximum flooding area of 1 ha and average depth of ~ 0.5 m. The minimum distance between each pair of wetlands was ~10 km. All wetlands had either one or two predominant habitat types (emergent and/or floating vegetation) and harbored fishes and tadpoles (Pires et al. 2018).

## Field methods and laboratory procedures

Invertebrates were collected in the austral spring (from September to October), the period of the year matching the mid-to-late wet phases of the wetland hydrological cycles (Bertuzzi et al. 2019; Knauth et al. 2019). Four samples were taken in the littoral zone of each wetland with a dip-net frame (width = 30 cm; mesh = 250 μm). Each sample consisted of a 1-m sweep on the water column after kicking up the substrate. The sampled material was preserved in situ with 10% formaldehyde. In the laboratory, invertebrate specimens were screened and identified under a stereomicroscope to the genus level whenever possible after consultation to bibliography (Fernández & Domínguez 2001, Merrit et al. 2008). The specimens were subsequently fixed in 80% alcohol and are archived at the invertebrate collection of the Laboratory of Ecology and Conservation of Aquatic Ecosystems at UNISINOS.

## References

Alvares, C.A., Stape, J.L., Sentelhas, P.C., De Moraes Gonçalves, J.L., Sparovek, G. 2013. Köppen’s climate classification map for Brazil. Meteorol. Zeitschrift, 22, 711–728.

Bertuzzi, T., Pires, M.M., Maltchik, L. 2019. Drivers of the beta diversity of aquatic plant communities along a latitudinal gradient in southern Brazilian coastal ponds. J. Veg. Sci. 30, 281–290.

Fernández, H., Domínguez, E. 2001. Guía para la determinación de los artrópodos bentónicos sudamericanos, first ed. Universidad Nacional de Tucumán, Instituto Miguel Lillo, Tucumán.

Knauth, D.S., Pires, M.M., Stenert, C., Maltchik, L. 2019. Disentangling the role of niche-based and spatial processes on anuran beta diversity in temporary ponds along a forest-grassland transition. Aquat. Sci. 81, e63.

Merritt, R., Cummins, K., Berg, M.B. 2008. An Introduction to the Aquatic Insects of North America, fifth ed. Kendall/Hunt, Dubuque, Iowa.

Maltchik, L., Costa, E.S., Becker, C.G., Oliveira, A.E. 2003. Inventory of wetlands of Rio Grande do Sul (Brazil). Pesquisas: Botânica, 53, 89–100.

Pires, M.M., Stenert, C., Maltchik, L. 2018. Drivers of beta diversity of Odonata along a forest-grassland transition in southern Brazilian coastal ponds. Freshw. Sci. 37, 357–366.

Villwock J.A., Tomazelli L.J., 2006. Planície Costeira do Rio Grande do Sul: gênese e paisagem atual, in: Becker, F.G., Ramos, R.A., Moura, L.A. (Eds.), Biodiversidade; Regiões da Lagoa do Casamento e dos Butiazais de Tapes, planície costeira do Rio Grande do Sul. Ministério do Meio Ambiente, Brasília, pp. 20–33.

# 6. Argentinean Patagonia (province of Chubut) *(modified from Epele et al. 2019)*

## Study region

The study area is located between 42°–44° S and 70°–71° W (28,600 km^2^) (Patagonia, Argentina), in the transition zone between Sub-Antarctic Forest and the Patagonian Steppe phytogeographical regions. These distinct regions are the result of the West–East rainfall gradient (Paruelo et al. 1998). The 26 studied wetlands were located in the temperate Patagonia at NW of Chubut Province (Argentina), where wetland pasturelands are very significant as water sources and sustain large livestock loads. All *mallines* (local name for wetlands) were used as pasturelands for a mix of livestock types (sheep, cows and horses). Access of livestock to mallines was unrestricted at all sites. Sampled wetlands were chosen according to their accessibility and similarity in land use (pastures), and were all geographically isolated. All sites were distant more than 5 km from urban areas. Surveys were conducted once in early austral summer (December 2006).

## Invertebrate collection and laboratory procedures

Pond aquatic invertebrates were sampled using a D-frame net (800 μm mesh). The net was horizontally (1.5 m) swept eight times, from the margins to the middle part of the ponds, removing invertebrates associated with epibenthos, nekton and pleuston. Contents of the 8 sweeps were pooled into 1 composite sample. Three composite samples were collected per site. Invertebrates were fixed in situ in 5% formalin. In the laboratory, samples were sorted and counted under 5 x magnification, and finally stored in 70% ethyl alcohol.

## References

Epele, L.B., Brand, C., Miserendino, M.L. 2019. Ecological drivers of alpha and beta diversity of freshwater invertebrates in arid and semiarid Patagonia (Argentina). Sci. Total Environ. 678, 62–73.

Paruelo, J.M., Jobbágy, E.G., Sala, O.E. 1998. Biozones of Patagonia (Argentina). Ecol. Austral, 8, 145–153.
