# Supplementary material for: Does taxonomic and numerical resolution affect the assessment of invertebrate community structure in New World freshwater wetlands?
Source: Ecol Indic. Author manuscript; Available in PMC 2021 Jun 1. (PMC7963273; doi:10.1016/j.ecolind.2021.107437)
Supplement: 2 [file NIHMS1668784-supplement-2.doc]

**Pires et al. 2020. Does taxonomic and numerical resolution affect the assessment of invertebrate community structure in New World freshwater wetlands? Ecol. Indicat. submitted.**

**Supporting information 2.** Numerical outputs of the linear correlations between richness and equitability of invertebrate communities calculated according to different numerical resolutions in each regions.

| Metric | Richness | | Shannon | |
| --- | --- | --- | --- | --- |
| Region | *r* | *P* | *r* | *P* |
| Northern US | 0.92 | 0.0001 | 0.88 | 0.0001 |
| Northeastern US | 0.83 | 0.0001 | 0.76 | 0.0001 |
| Western US | 0.93 | 0.0001 | 0.99 | 0.0001 |
| Southeastern US | 0.92 | 0.0001 | 0.93 | 0.0001 |
| Southern Brazil | 0.93 | 0.0001 | 0.97 | 0.0001 |
| Argentinian Patagonia | 0.89 | 0.0001 | 0.94 | 0.0001 |
