# Supplementary material for: Does taxonomic and numerical resolution affect the assessment of invertebrate community structure in New World freshwater wetlands?
Source: Ecol Indic. Author manuscript; Available in PMC 2021 Jun 1. (PMC7963273; doi:10.1016/j.ecolind.2021.107437)
Supplement: 3 [file NIHMS1668784-supplement-3.doc]

**Pires et al. 2020. Does taxonomic and numerical resolution affect the assessment of invertebrate community structure in New World freshwater wetlands? Ecol. Indicat. submitted.**

**Supporting information 3.** Numerical outputs of the beta diversity metrics (absolute values of total beta diversity; and of the turnover and nestedness components) across numerical and taxonomic resolutions. Abbreviations: Total beta = beta diversity calculated as total variance in a data set (according to Legendre & De Cáceres 2013). PA = presence-absence; RA = relative abundance; Turn = turnover component of beta diversity; Nest = nestedness component of beta diversity.

| **Region** | **Numerical resolution** | **Taxonomic  resolution** | | | | | |
| --- | --- | --- | --- | --- | --- | --- | --- |
|  |  | **Family** | | | **Lowest** | | |
|  |  | **Total beta** | **Turn** | **Nest** | **Total beta** | **Turn** | **Nest** |
| **Northern US** | **PA** | 0.162 | 0.093 | 0.069 | 0.208 | 0.125 | 0.082 |
|  | **RA** | 0.322 | 0.223 | 0.100 | 0.347 | 0.257 | 0.090 |
| **Northeastern US** | **PA** | 0.233 | 0.118 | 0.115 | 0.313 | 0.190 | 0.124 |
|  | **RA** | 0.374 | 0.108 | 0.267 | 0.421 | 0.155 | 0.266 |
| **Western US** | **PA** | 0.222 | 0.104 | 0.118 | 0.247 | 0.127 | 0.120 |
|  | **RA** | 0.374 | 0.085 | 0.287 | 0.377 | 0.089 | 0.237 |
| **Southeastern US** | **PA** | 0.191 | 0.106 | 0.085 | 0.232 | 0.149 | 0.083 |
|  | **RA** | 0.294 | 0.158 | 0.136 | 0.303 | 0.167 | 0.136 |
| **Southern Brazil** | **PA** | 0.177 | 0.096 | 0.081 | 0.225 | 0.140 | 0.085 |
|  | **RA** | 0.382 | 0.125 | 0.257 | 0.391 | 0.134 | 0.257 |
| **Argentinean Patagonia** | **PA** | 0.240 | 0.154 | 0.086 | 0.337 | 0.235 | 0.102 |
|  | **RA** | 0.409 | 0.142 | 0.300 | 0.436 | 0.170 | 0.270 |
