# Supplementary material for: Does taxonomic and numerical resolution affect the assessment of invertebrate community structure in New World freshwater wetlands?
Source: Ecol Indic. Author manuscript; Available in PMC 2021 Jun 1. (PMC7963273; doi:10.1016/j.ecolind.2021.107437)
Supplement: 4 [file NIHMS1668784-supplement-4.doc]

**Supporting Information 4.** Numerical outputs of the paired t-tests for the absolute values and relative contribution of the turnover and nestedness fractions of beta diversity between the original invertebrate composition data sets calculated according to different taxonomic and numerical resolutions. PA = presence-absence; RA = relative abundance; ‘Family’ = family-level taxonomic resolution; ‘Highest’ = highest possible taxonomic level.

| **Metric** | **Total_beta** | | **Turnover (%)** | | **Nestedness (%)** | |
| --- | --- | --- | --- | --- | --- | --- |
| **Approach** | **T-value** | **p** | **T-value** | **p** | **T-value** | **p** |
| Family - PA vs. Family - RA | 11.315 | <0.001 | 2.243 | 0.074 | -2.243 | 0.074 |
| Highest - PA vs. Highest - RA | 8.733 | <0.001 | 2.514 | 0.053 | -2.514 | 0.053 |
| Family - PA vs. Highest - PA | 5.122 | 0.003 | 5.733 | 0.002 | -5.733 | 0.002 |
| Family - RA vs. Highest - RA | 2.999 | 0.03 | 2.988 | 0.03 | -2.988 | 0.03 |
