# Supplementary material for: Does taxonomic and numerical resolution affect the assessment of invertebrate community structure in New World freshwater wetlands?
Source: Ecol Indic. Author manuscript; Available in PMC 2021 Jun 1. (PMC7963273; doi:10.1016/j.ecolind.2021.107437)

**Pires et al. 2020. Does taxonomic and numerical resolution affect the assessment of invertebrate community structure in New World freshwater wetlands? Ecol. Indicat. submitted.**

**Supporting information 4.** Procrustes rotation plots between the original invertebrate composition data sets calculated according to different taxonomic and numerical resolutions. Abbreviations in the bottom-right legend box: PA = presence-absence; RA = relative abundance; ‘Family’ = family-level taxonomic resolution; ‘Lowest’ = lowest level practical taxonomic resolution.


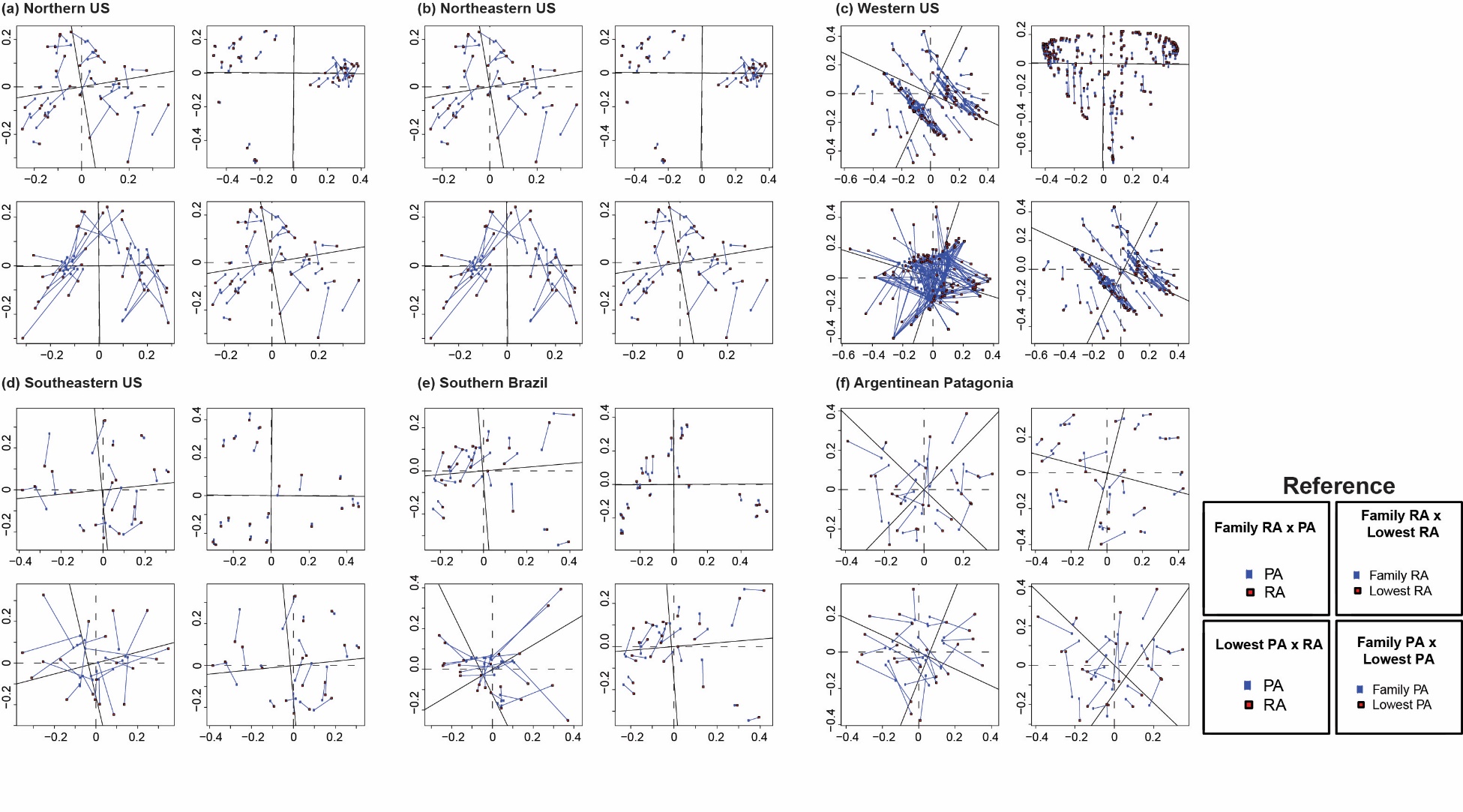

Supplement: 5 [file NIHMS1668784-supplement-5.doc]
